# S100b in acute ischemic stroke clots is a biomarker for post-thrombectomy intracranial hemorrhages

**DOI:** 10.3389/fneur.2022.1067215

**Published:** 2023-01-23

**Authors:** Rosanna Rossi, Andrew Douglas, Sara Molina Gil, Duaa Jabrah, Abhay Pandit, Michael Gilvarry, Ray McCarthy, James Prendergast, Katarina Jood, Petra Redfors, Annika Nordanstig, Erik Ceder, Dennis Dunker, Jeanette Carlqvist, István Szikora, John Thornton, Georgios Tsivgoulis, Klearchos Psychogios, Turgut Tatlisumak, Alexandros Rentzos, Karen M. Doyle

**Affiliations:** ^1^Department of Physiology and Galway Neuroscience Centre, School of Medicine, National University of Ireland, Galway, Ireland; ^2^CÚRAM–SFI Research Centre in Medical Devices, National University of Ireland Galway, Galway, Ireland; ^3^Cerenovus, Galway, Ireland; ^4^Department of Neurology, Sahlgrenska University Hospital, Gothenburg, Sweden; ^5^Department of Clinical Neuroscience, Institute of Neuroscience and Physiology, Sahlgrenska Academy at University of Gothenburg, Gothenburg, Sweden; ^6^Department of Interventional and Diagnostic Neuroradiology, Sahlgrenska University Hospital, University of Gothenburg, Gothenburg, Sweden; ^7^Department of Neurointerventions, National Institute of Clinical Neurosciences, Budapest, Hungary; ^8^Department of Radiology, Royal College of Surgeons in Ireland, Beaumont Hospital, Dublin, Ireland; ^9^Second Department of Neurology, “Attikon” University Hospital, National and Kapodistrian University of Athens, Athens, Greece; ^10^Stroke Unit, Metropolitan Hospital, Piraeus, Greece

**Keywords:** S100b, stroke biomarkers, thrombus, acute ischemic stroke, post-thrombectomy intracranial hemorrhages

## Abstract

**Background and purpose:**

Post-thrombectomy intracranial hemorrhages (PTIH) are dangerous complications of acute ischemic stroke (AIS) following mechanical thrombectomy. We aimed to investigate if S100b levels in AIS clots removed by mechanical thrombectomy correlated to increased risk of PTIH.

**Methods:**

We analyzed 122 thrombi from 80 AIS patients in the RESTORE Registry of AIS clots, selecting an equal number of patients having been pre-treated or not with rtPA (40 each group). Within each subgroup, 20 patients had developed PTIH and 20 patients showed no signs of hemorrhage. Gross photos of each clot were taken and extracted clot area (ECA) was measured using ImageJ. Immunohistochemistry for S100b was performed and Orbit Image Analysis was used for quantification. Immunofluorescence was performed to investigate co-localization between S100b and T-lymphocytes, neutrophils and macrophages. Chi-square or Kruskal-Wallis test were used for statistical analysis.

**Results:**

PTIH was associated with higher S100b levels in clots (0.33 [0.08–0.85] vs. 0.07 [0.02–0.27] mm^2^, H1 = 6.021, *P* = 0.014^*^), but S100b levels were not significantly affected by acute thrombolytic treatment (*P* = 0.386). PTIH was also associated with patients having higher NIHSS at admission (20.0 [17.0–23.0] vs. 14.0 [10.5–19.0], H1 = 8.006, *P* = 0.005) and higher number of passes during thrombectomy (2 [1–4] vs. 1 [1–2.5], H1 = 5.995, *P* = 0.014^*^). S100b co-localized with neutrophils, macrophages and with T-lymphocytes in the clots.

**Conclusions:**

Higher S100b expression in AIS clots, higher NIHSS at admission and higher number of passes during thrombectomy are all associated with PTIH. Further investigation of S100b expression in AIS clots by neutrophils, macrophages and T-lymphocytes could provide insight into the role of S100b in thromboinflammation.

## Introduction

Post-thrombectomy intracranial hemorrhages (PTIH) are the most serious complication of endovascular procedures following acute ischemic stroke (AIS). Intracranial hemorrhage can take a wide range of different forms, including extraparenchymal (subdural hematoma and subarachnoid hemorrhage) and intraparenchymal (1). Intracranial hemorrhage occurs when the blood-brain barrier (BBB) is sufficiently damaged to permit extravasation of blood components into the brain parenchyma, increasing stroke morbidity and mortality (2). There are several factors associated with increased risk of PTIH, such as stroke severity (3), recanalization therapy (both thrombolysis and thrombectomy) (4), hypertension (5), hyperglycemia (3, 5) and age (6).

In the last few years, there have been several efforts to predict intracranial hemorrhage after AIS (3, 6–9). Also, many studies looking for novel biomarkers for stroke diagnosis and prognosis have converged on the crucial role of inflammation (8), focusing on a panel of proteins potentially useful for this purpose (10–12). Several proteins have been explored as potential biomarkers of hemorrhagic transformation after acute ischemic stroke, including matrix metalloproteinase-9 (MMP9), neuron-specific enolase (NSE), cellular-fibronectin (c-Fn), plasminogen activator inhibitor (PAI-1), thrombin-activated fibrinolysis inhibitor (TAFI) and S100b (10), which was the main focus of this manuscript. The glial protein S100b can be produced by several peripheral cell subtypes (13) including T-lymphocytes (14, 15) and it is not a specific indicator for stroke, as its levels are increased also in other neurological conditions (16). However, it is among the most interesting candidates that have been investigated as stroke biomarkers, showing potential in discriminating between ischemic and hemorrhagic stroke (17, 18). Nonetheless, there is evidence that increased levels of S100b in blood of AIS patients are associated with increased intracranial hemorrhage rate following thrombolytic therapy (19). However, the involvement of S100b in stroke has not yet been fully investigated.

We evaluated the expression of S100b in 122 thrombi retrieved from 80 AIS patients, with equal numbers with or without acute thrombolytic administration. In this study, we investigated if there was a difference in S100b expression in AIS clots extracted from patients with or without PTIH, to further explore the role of S100b as a possible biomarker for PTIH. Furthermore, the possibility that white blood cell subtypes are the source of S100b in AIS clots was investigated.

## Materials and methods

### Patient cohort

**Figure 1:**
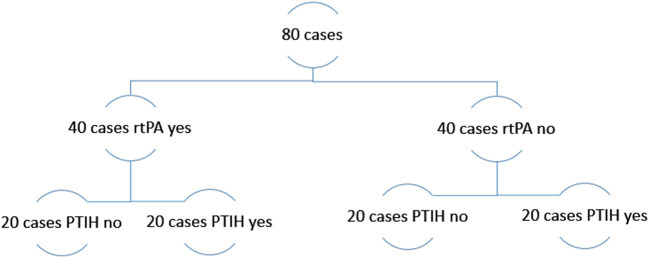
Experimental plan of the study.

Eighty acute ischemic stroke cases from the RESTORE registry of AIS clots were included in this study. The RESTORE registry is registry of thrombotic material extracted *via* mechanical thrombectomy from 1,000 AIS patients during the period February 2018 to December 2019 from four stroke centers in Europe (20). Two of the four participating hospitals (Sahlgrenska University Hospital, Gothenburg and Metropolitan Hospital, Athens) provided information on PTIH (472 patients). Of these, 81 patients developed PTIH, i.e., 17%. In this study, we analyzed clot samples from an equal number of cases in the two subgroups PTIH yes and PTIH no, closely matched for factors such as age, sex, etiology, and thrombolysis yes/no. PTIH was identified by two experienced radiologists at each clinical site on a CT scan 24-36h after thrombectomy and classified according to the European Cooperative Acute Stroke Study II (ECASS II) classification system (21). The experimental plan is illustrated in Figure 1. This study was conducted in accordance with the ethical standards of the Declaration of Helsinki and its amendments (22), by approval of the regional hospital ethics committees and National University of Ireland Galway research ethics committees (16-SEPT-08). We included only patients >18 years, having been treated with mechanical thrombectomy for AIS whose thrombus material was available for analysis and having information whether the patients suffered (or not) PTIH. For each patient we collected an anonymized data abstraction form containing pertinent procedural data, such as rtPA administration, NIHSS score at admission, occlusion location, stroke etiology, number of passes for clot removal, final mTICI score and hemorrhagic transformation incidence. Suspected stroke etiology was reported according to the TOAST classification system (23). As it has been reported that rtPA administration may be associated with a higher risk of hemorrhagic transformation following AIS, therefore we included equal numbers (*n* = 40) of patients treated with bridging-therapy (rtPA and mechanical thrombectomy) and 40 patients treated with mechanical thrombectomy alone. For each subgroup we included equal numbers (*n* = 20) of patients with PTIH after mechanical thrombectomy and 20 closely matched controls without PTIH. Controls were AIS patients treated with mechanical thrombectomy but with no sign of hemorrhage and matched as closely as possible to the PTIH yes subgroup for age, sex, etiology and thrombolysis yes/no.

### Thrombi collection, size measurement and processing

Thrombotic material extracted *via* mechanical thrombectomy was collected *per pass* at the hospital venue in separate pots containing 10% formalin and shipped to NUI Galway. A gross photo of each thrombus was taken with a Canon EOS 1300D Camera and the relevant Extracted Clot Area (ECA) was measured and used as an estimate of the extracted clot size, by drawing around each fragment with a specific tool using ImageJ software, as previously reported (24–27). In brief, to measure the Extracted Clot Area (ECA), the gross photo of the extracted clot was opened using ImageJ software, the scale was set and the Polygon tool was used to draw a region of interest around each clot fragment in the gross photo, and summed to give overall ECA for the sample. Following gross photos, thrombi were placed in histological cassettes for tissue processing and paraffin embedding. We analyzed a total of 122 thrombi, collected per pass from the 80 cases in this study.

### Immunohistochemistry staining

After paraffin embedding, 3 μm sections were cut from each block with a microtome and S100b staining was performed by Immunohistochemistry (IHC) on a Leica Bond-III autostainer using a BOND Polymer Refine Red Detection kit (Leica Biosystems #DS9390). Antigen retrieval with tris-EDTA (Leica Biosystems #AR9640) was performed for 10 min. Primary antibody rabbit anti-S100b (abcam, ab41548, 1:100 dilution) incubation time was 15 min, followed by 30 min of incubation with an anti-rabbit secondary antibody. Counterstaining of tissue using haematoxylin was performed for 5 min. Sections were then washed with a washing solution (Leica Biosystems #AR9590) and rinsed in distilled water. Sections were then dehydrated in alcohol, cleared in xylene, and mounted with DPX. Negative controls were performed by omission of the primary antibody step. Entorhinal cortical brain tissue (BioIVT) was used as positive control tissue for S100b expression.

### Slide scanning and quantification

Immunohistochemically stained slides were scanned on an Olympus vs120 slide scanner at 20× magnification and digital whole slide scan images were generated. Quantification was performed on the digital slides using Orbit Image Analysis Software (www.orbit.bio) (28), as previously described (29). In brief, we created exclusion and inclusion models to distinguish regions to be excluded (e.g., background and artifact) and regions containing the component of interest, S100b, enabling its quantitative assessment within each clot.

We quantified the expression of S100b in each case as area (in mm^2^) by multiplying the component percent by the relevant ECA. For cases involving multiple passes, we summed the values of S100bXECA for all passes.

### Immunofluorescence

Immunofluorescence staining was performed on a subset of samples in order to evaluate co-localization of S100b and three WBC markers, respectively CD3 staining for T-lymphocytes, CD68 staining for macrophages and CD66b staining for neutrophils. We used inflamed tonsil tissue (BioIVT), as positive control tissue for CD3, CD68, and CD66b. The primary antibodies used were the following: rabbit anti-S100b (abcam ab41548, 1:100 dilution); mouse anti-CD3 (abcam ab17143, 1:10); mouse anti-CD68 (abcam ab955, 1:50) mouse anti-CD66b (Novus biological NB100-77808, 1:100). Secondary antibodies were: Goat anti-mouse IgG H&L (Alexa Fluor 594, abcam ab150116, 1:200) and Goat anti-rabbit IgG H&L, (Alexa Fluor 488, abcam ab150077, 1:200).

After deparaffinization with xylene and following rehydration with 100, 95, 70 and 50% alcohol, 3 μm sections of thrombus tissue were incubated for 20 min with Tris-EDTA buffer in a microwave at 98°C. Sections were washed with phosphate-buffered saline (PBS) followed by PBS containing 0.2% Tween 20 (PBS-Tx) and incubated with blocking buffer (3% normal goat serum, NGS, in PBS-Tx) for 1 h at room temperature under agitation. Incubation with S100b and one of the WBC primary antibodies per slide followed for 1 h at 37°C, then over night at 4°C. After washing, sections were incubated with secondary antibodies for 1 h at 37°C and then cover slipped with 4',6-diamidino-2-phenylindole (DAPI) mounting medium for nucleic acid staining.

Immunostaining images were captured by using the objective of 60× in a FV3000 Confocal Laser Scanning microscope (Olympus) and analyzed with FIJI software (ImageJ).

### Statistical analysis

Statistical analysis was performed with IBM SPSS-25 software and graphs were created with GraphPad Prism 9.2.0. Quantitative variables did not follow a standard normal distribution as indicated by Kolmogorov-Smirnov test. Therefore, the Chi square test or Kruskal-Wallis test were used to assess statistically significant differences among the groups, respectively for nominal or continuous variables. Correlation analysis was also performed (Spearman's Rho). The level of statistical significance was set at *p* < 0.05 (two-sided). Results are reported as median [IQ1–IQ3] or number and percentage (%) of cases.

## Results

### Baseline characteristics of the patients

Baseline clinical and procedural characteristics of the 80 patients selected are reported in Table 1, for the overall population analyzed and according to whether PTIH occurred or not. Main types of PTIH defined according to ECASS II classification are the following: small petechial haemorrhagic infarction (HI1), confluent petechial haemorrhagic infarction (HI2), small parenchymal hemorrhage (PH1) (<30% of infarct, mild mass effect), and large parenchymal hemorrhage (PH2, >30% of infarct, marked mass effect). In our cohort we found 14 cases of HI1 (35% of HT), 7 cases of HI2 (17.5% of HT), 7 cases of PH1 (17.5%) and 7 cases of PH2 (17.5%). We also found 4 cases (10%) of subarachnoid hemorrhage (SAH) and 1 case (2.5%) of subdural hematoma (SDH). There was no significant difference between the two subgroups in terms of sex (*P* = 0.822), age (*P* = 8.885), stroke etiology (*P* = 0.966) and occlusion location (*P* = 0.461). Also, no difference was found in terms of onset to groin puncture time (*P* = 0.787), onset to recanalization time (*P* = 0.953) and final mTICI score (*P* = 0.086). However, patients with no signs of hemorrhage had lower rate of mTICI 2b (17.5 vs. 32.5%) and higher rate of mTICI 3 (52.5 vs. 30%) compared to PTIH yes subgroup. NIHSS at admission was significantly higher for PTIH yes subgroup (*P* = 0.005^*^). The total number of passes for clot removal was also significantly higher for the PTIH yes subgroup (*P* = 0.014^*^); Table 1.

**Table 1:** Baseline clinical and procedural characteristics of the overall cohort of patients and divided according whether they had or not post-thrombectomy intracranial hemorrhage (PTIH).

**Sex**	**Overall cohort of patients (*N* = 80)**	**PTIH YES (*N* = 40)**	**PTIH NO (*N* = 40)**	**Statistical analysis**
Male	45 (56.3%)	23 (57.5%)	22 (55.0%)	*X*^1^ *=* 0.051, *P* = 0.822
Female	35 (43.8%)	17 (42.5%)	18 (45.0%)	
Age (years)	75.0 [64.5–83.0]	75.0 [63.0–82.0]	74.5 [65.5–83.0]	H1 = 0.021, *P* = 0.885
**Stroke etiology**				
Patients with cardioembolic etiology	32 (40%)	15 (37.5%)	17 (42.5%)	*X*^3^ *=* 0.268, *P* = 0.966
Patients with large artery atherosclerosis etiology	16 (20%)	8 (20.0%)	8 (20.0%)	
Patients with other etiology^a^	4 (5%)	2 (5.0%)	2 (5.0%)	
Patients with cryptogenic etiology	28 (35%)	15 (37.5%)	13 (32.5%)	
NIHSS admission	18.0 [11.0–21.5]	20.0 [17.0–23.0]	14.0 [10.5–19.0]	H1 = 8.006, *P* = 0.005^*^
**Occluded vessel (s)** ^**b**^				
MCA, M1	31 (38.8%)	14 (35.0%)	17 (42.5%)	H1 = 0.543, *P* = 0.461
MCA, M2	8 (10.0%)	4 (10.0%)	4 (10.0%)	
MCA, M3	1 (1.3%)	0 (0.0%)	1 (2.5%)	
MCA (multiple branches/segments)	6 (7.5%)	3 (7.5%)	3 (7.5%)	
ICA&ICA terminus	14 (17.5%)	7 (17.5%)	7 (17.5%)	
ACA	1 (1.3%)	1 (2.5%)	0 (0.0%)	
VB	6 (7.5%)	4 (10.0%)	2 (5.0%)	
PCA	1 (1.3%)	0 (0.0%)	1 (2.5%)	
Tandem occlusion	6 (7.5%)	4 (10.0%)	2 (5.0%)	
Other dual	1 (1.3%)	1 (2.5%)	0 (0.0%)	
3 or more occluded vessels	5 (6.3%)	2 (5.0%)	3 (7.5%)	
Median number of passes performed during the endovascular procedure	2 [1–3]	2 [1–4]	1 [1–2.5]	H_1_ = 5.995, *P* = 0.014^*^
Onset to groin puncture time (minutes)	145 [53–265]	145 [45–300]	142.5 [54.5–262.5]	H1 = 0.073, *P* = 0.787
Onset to recanalization time (minutes)	236 [88–345]	235 [99–355]	258 [88–328]	H1 = 0.003, *P* = 0.953
**Final mTICI score**				
mTICI 0	4 (5.0%)	2 (5.0%)	2 (5.0%)	H1 = 2.948, *P* = 0.086
mTICI 1	1 (1.3%)	0 (0.0%)	1 (2.5%)	
mTICI 2a	8 (10.0%)	5 (12.5%)	3 (7.5%)	
mTICI 2b	20 (25.0%)	13 (32.5%)	7 (17.5%)	
mTICI 2c	14 (17.5%)	8 (20.0%)	6 (15.0%)	
mTICI 3	33 (41.3%)	12 (30.0%)	21 (52.5%)	

^a^Other etiology included arterial dissection.

^b^MCA, Middle Cerebral Artery; ICA, Internal Carotid Artery; ACA, Anterior Cerebral Artery; VB, Vertebro-basilar; PCA, Posterior Cerebral Artery.

Data given as *N* (%) of cases or median [IQ1, IQ3].

^*^Statistically significant.

### S100b expression in AIS clots is associated with WBC

**Figure 2:**
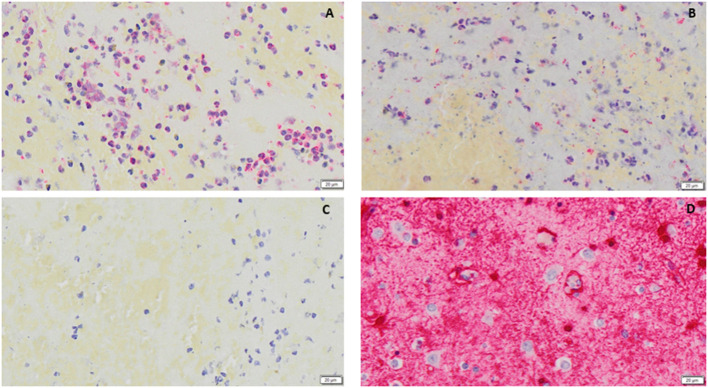
**(A, B)** examples of S100b (red immunostaining) in clots, respectively from a patient with **(A)** and without **(B)** Post-thrombectomy Intracranial Hemorrhage are shown. In **(C)** a negative control is shown, while in **(D)** positive control tissue (human entorhinal brain cortex) is shown. Nuclei are stained blue (counterstaining with haematoxylin). All images were captured using the 20× objective (scale bar 20 μm).

We distinguished clots with different expression of S100b (Figure 2). Interestingly, as depicted in panels A–B, we noticed that S100b was closely associated with nucleated cells in the clots, leading us to perform further analysis with immunofluorescence co-staining to assess sub-types of WBC associated with S100b expression.

### S100b expression in AIS clots is higher in clots from patients with PTIH regardless of rtPA administration preceding mechanical thrombectomy, NIHSS on admission and age

**Figure 3:**
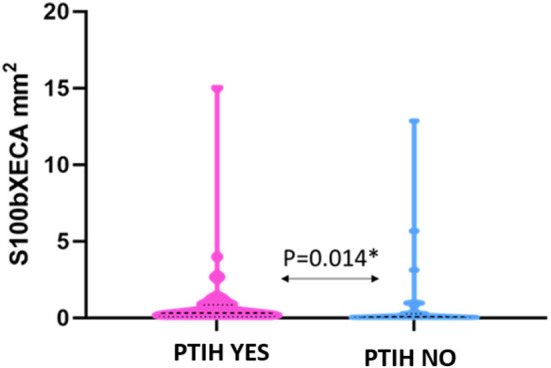
Violin plot showing that S100b expression in clots from patients with PTIH is significantly higher than patients without signs of hemorrhage. Dashed lines represent the median while dotted lines represent the interquartile ranges, Q1 **(lower dotted lines)** and Q3 **(upper dotted lines)**. *Statistically significant.

S100b expression in clots from patients with PTIH was statistically significantly higher than those from patients without HT (0.33 [0.08–0.85] vs. 0.07 [0.02–0.27] mm^2^, *P* = 0.014, when expressed as area, Figure 3. A similar trend was apparent when S100b was expressed as overall percentage (0.53 [0.21–1.48]% for PTIH yes vs. 0.32 [0.09–0.80]% for PTIH no, although not statistically different (*P* = 0.100).

We did not find any significant difference between expression of S100b in clots from patients pre-treated with rtPA and those of patients treated with mechanical thrombectomy alone (*P* = 0.386). Additionally, we did not find any significant correlation between S100b levels and NIHSS on admission (Spearman's rho = 0.213, *P* = 0.058), or age (Spearman's rho = −0.120, *P* = 0.290). The different types of hemorrhagic transformation observed and S100b expression in extracted thrombus material is described in Supplementary Table 1. We did not find any statistically significant difference in terms of S100b expression among the several types of PTIH, although we acknowledge that S100b expression in PH was higher than in HI. This could be worthy of further future investigation.

### S100b expression is associated with macrophages, neutrophils and T-lymphocytes in clots

**Figure 4:**
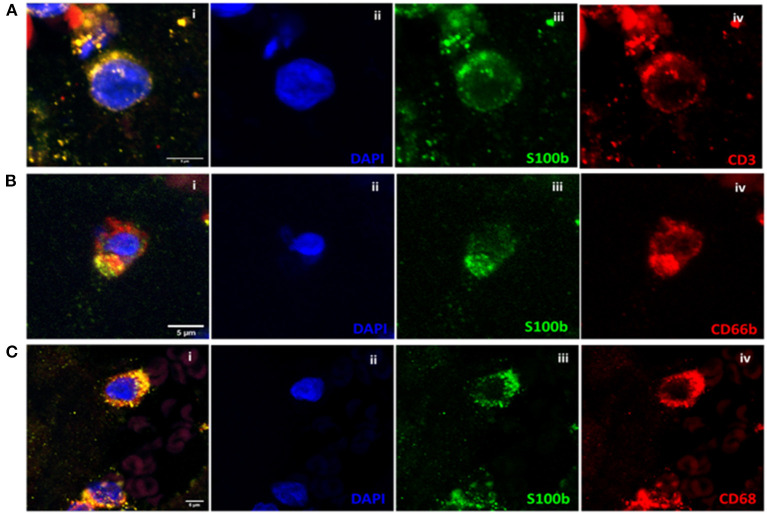
**(A)** Co-localization analysis of S100b and CD3 in clot with the three channels together (i) and separately (ii–iv). **(B)** Co-localization analysis of S100b and CD66b in clot with the three channels together (i) and separately (ii–iv). **(C)** Co-localization analysis of S100b and CD68 in clot with the three channels together (i) and separately (ii–iv). All images were captured using the 60× objective (scale bar 5 μm).

Immunofluorescence staining revealed association of S100b with the three WBC subtypes we studied, i.e., T-lymphocytes (CD3), Figure 4A, neutrophils (CD66b), Figure 4B and with macrophages (CD68), Figure 4C.

## Discussion

Emergent reperfusion therapy is the cornerstone of treatment in AIS, aiming to restore cerebral blood flow to salvageable ischemic tissue to reduce patient disability. However, PTIH is the most feared complication following endovascular procedures (30). Hemorrhagic infarction following arterial thrombosis and embolism has been suggested as a natural progression of ischemic stroke (31–34). The incidence of this phenomenon greatly varies depending on several risk factors (3–6) and has been reported to range from 0 to 85% in different studies (35).

Severe stroke, expressed as high NIHSS score at admission, is generally associated with a higher probability of intracranial hemorrhage (3, 8), which is in line with the findings in the present study. Both thrombolysis and mechanical thrombectomy have been associated with increased risk of intracranial hemorrhage (4). According to some studies, intracranial hemorrhage following thrombolytic administration can be directly linked to rtPA activity, resulting from reperfusion of cerebral vessels whose integrity has been disrupted by severe ischemia (36, 37). It has been also suggested that alteplase may promote intracranial hemorrhage through non-fibrinolytic mechanisms, such as activation of the immune system (38), neutrophil degranulation and release of matrix metalloproteinase-9 (MMP-9) (39) favoring BBB disruption (40). Mechanical thrombectomy may increase intracranial hemorrhage rate due to direct vessel wall damage during endovascular procedures. The degree of damage has been shown to be proportional to the number of passes required for clot retrieval (41). Our results support these findings, as we observed a significantly higher number of passes during mechanical thrombectomy in the cases with PTIH.

Due to the high morbidity and mortality associated with PTIH, many efforts have been made to find new biomarkers and predictors of intracranial hemorrhage following AIS (3, 6–9). In this regard, S100b might have some potential. S100b is a calcium-binding protein belonging to the S100 family, which comprises more than 20 family members (13). At nanomolar concentrations, S100b has been shown to promote neurite outgrowth in cerebral cortex neurons *in vitro* and to enhance survival of neurons during development (42), neuronal maturation and to stimulate glial cell proliferation (43). Furthermore, S100b reduces cell death and protects against mitochondrial loss of function resulting from glucose deprivation (42, 44). However, at micromolar concentrations, S100b can cause deleterious effects. At these concentrations, it has been shown that extracellular S100b promotes its neurotoxic effects by stimulating the expression of proinflammatory cytokines and inducing apoptosis *in vitro* (44). Inflammation is a common characteristic of many neurological disorders. Elevated levels of S100b protein in biological fluids are observed in several neurological disorders, such as multiple sclerosis (45), Alzheimer's disease (46), Parkinson's disease (47), amyotrophic lateral sclerosis (48) and stroke (49).

S100b has been investigated as a possible biomarker to distinguish hemorrhagic stroke from ischemic stroke and some studies have shown that S100b concentrations in blood were higher for hemorrhagic stroke compared to ischemic stroke (50, 51). Also, a previous study highlighted how increased S100b levels in serum of AIS patients treated with thrombolysis might be a predictor of further hemorrhagic transformation (19). These results are in line with the findings of the present study. We have shown higher S100b expression in AIS thrombi from patients with PTIH compared to those from the non-PTIH group. We also demonstrated that S100b expression in clots is not significantly affected by acute thrombolytic administration.

Whether the original source of S100b in the clots is peripheral or of central origin is still unclear, but worthy of further study.

In this study, immunohistochemistry and Immunofluorescence staining revealed that S100b expression in clots was associated with WBC nuclei, CD68+, CD66b+, and CD3+. The association of S100b with phagocytic cells such as macrophages and neutrophils is interesting, since S100 proteins can work as Damage-Associated Molecular Pattern (DAMP) molecules (52). DAMPs are biomolecules that are released from damaged or stressed cells and could act as endogenous danger signals to induce a rapid inflammatory response (53). Furthermore, S100 proteins play an important role as regulators of macrophage-mediated inflammation (54). In particular, S100b can up-regulate macrophage production of pro-inflammatory cytokines and worsen severity of inflammation (55), therefore, we could hypothesize a similar role also in stroke inflammation. Association between S100b and neutrophils has also been observed since it is known that S100b induces neutrophil migration to sites of inflammation (56, 57). It has been shown that neutrophils can induce damage in the ischemic area by causing neuronal death, destruction of the BBB, and brain edema (58, 59). Neutrophil extracellular traps can further activate platelets and thrombotic processes (60).

Presence of S100b in T-lymphocytes was first detected by Kanamori et al. (61) in 1984. Further studies proved that a cytotoxic T subtype (CD3+ CD8+ CD16-) and a natural killer subtype (CD3- CD8- CD16+) of lymphocytes is able to produce S100b upon stimulation (14, 15). Our results are in line with these studies. The association of S100b with CD3+ lymphocyte subtypes suggests that this protein acts as an interface to immunological processes in various physiological and pathological conditions although further studies are necessary to better clarify its function. The connection between inflammation and thrombosis in cardiovascular diseases is becoming more and more evident. Immunothrombosis is activated in the setting of bacterial and viral infection. Targeting inflammation to prevent cardiovascular events is an emerging concept as it is known that inflammation increases thrombotic tendency. Main cellular drivers of this process are platelets and innate immune cells, primarily neutrophils and monocytes which interplay with platelets and flanked by the activation of the complement system promote coagulation (62). T-lymphocytes play a major role in the initiation and perpetuation of inflammatory cascades as well, involving crosstalk with other immune cells, especially by modulating macrophage response (63), although their specific role in thrombus formation is still unclear (64).

### Study limitations

Our study design focused on comparing two closely matched cohorts, respectively having, or not having experienced PTIH. We are aware that our cohort design approach may be prone to biases. A further study with a retrospective case control design, group comparisons adjusted for multiple testing and calculation of odds ratios would be useful. We did not assess S100b levels in serum in this study. S100b serum levels are known to be higher in stroke cases with larger lesion volumes (65, 66). It would be of interest to assess if clot S100b content reflects serum levels in future work. Also, as S100b levels can increase in cases of pre-stroke trauma or very recent surgery and this should be considered in further studies. Furthermore, it would be of interest to probe further if the source of S100b in the clots is astroglial or entirely extracerebral by using a second glial marker such as Glial Fibrillary Acidic Protein (GFAP) in future work. Finally, we did not take into account other factors that might influence occurrence of PTIH, such as pre-treatment with antiplatelet or anticoagulant medications and elevated blood pressure during and after the endovascular procedure. Also, because of the extensive thrombus heterogeneity, it is possible that thrombus composition might affect S100b expression. It would be of interest to consider these factors in further studies.

## Conclusion

From our observations, we can conclude that a higher expression of S100b in the retrieved clots is associated with PTIH regardless of thrombolytic administration. We also found other factors directly correlating with PTIH, such as higher NIHSS score at admission and higher number of passes during mechanical thrombectomy. Furthermore, from co-localization studies we observed that S100b in retrieved AIS clots was associated with macrophages, neutrophils and some T-lymphocytes, suggesting it may have an effect on thrombo-inflammatory activity, although we acknowledge that further investigation is necessary to confirm our results.

## Data availability statement

The original contributions presented in the study are included in the article/Supplementary material, further inquiries can be directed to the corresponding authors.

## Ethics statement

The studies involving human participants were reviewed and approved by the regional ethics committee of Gothenburg (approval numbers 233-17 and T017-18 dates 06-04-2017 and 16-01-2018 respectively), the 'Epitropi Iatrikis Ithikis Kai Deontologias Therapeutiriou Metropolitan' - Metropolitan Hospital Ethics Comittee (approval number 2430, 01-10-2018) and by the National University of Ireland Galway Research Ethics Committees (16-SEPT-08) following the ethical standards of the Declaration of Helsinki and its amendments. The patients/participants provided their written informed consent to participate in this study.

## Author contributions

KD obtained the funds for the study, coordinated study design implementation and supervised the writing of the manuscript. RR participated in samples collection, developed the study design, composed the manuscript, performed the IHC and the IF analysis, performed the statistical analysis and wrote the results and discussion. AD participated in samples collection, performed IHC quantification and IF staining. SG performed the ECA measurements, the IF staining and the confocal analysis. JP and DJ participated in the sample collection and in the slide scanning and quantification. KJ, PR, AN, EC, DD, JC, GT, KP, and AR performed the thrombectomy at the several hospitals, collected samples and procedural data. IS, JT, KP, GT, TT, and AR contributed to the study design and were responsible thrombus collection at the relevant stroke center. AP, MG, and RM contributed to develop the study design and funding acquisition. All the authors have read and reviewed the manuscript.

## References

[1] Charbonnier G Bonnet L Biondi A Moulin T. Intracranial bleeding after reperfusion therapy in acute ischemic stroke. Front Neurol. (2021) 11:629920. 10.3389/fneur.2020.629920 33633661 PMC7900408

[2] van Kranendonk KR Treurniet KM Boers AMM Berkhemer OA van den Berg LA Chalos V. Hemorrhagic transformation is associated with poor functional outcome in patients with acute ischemic stroke due to a large vessel occlusion. J Neurointerv Surg. (2019) 11:464–8. 10.1136/neurintsurg-2018-014141 30297537

[3] Kidwell CS Saver JL Carneado J Sayre J Starkman S Duckwiler G. Predictors of hemorrhagic transformation in patients receiving intra-arterial thrombolysis. Stroke. (2002) 33:717–24. 10.1161/hs0302.104110 11872894

[4] Jickling GC Liu D Stamova B Ander BP Zhan X Lu A. Hemorrhagic transformation after ischemic stroke in animals and humans. J Cereb Blood Flow Metab. (2014) 34:185–99. 10.1038/jcbfm.2013.203 24281743 PMC3915212

[5] Kerenyi L Kardos L Szász J Szatmári S Bereczki D Hegedüs K. Factors influencing hemorrhagic transformation in ischemic stroke: a clinicopathological comparison. Eur J Neurol. (2006) 13:1251–5. 10.1111/j.1468-1331.2006.01489.x 17038041

[6] Mazya M Egido JA Ford GA Lees KR Mikulik R Toni D. Predicting the risk of symptomatic intracerebral hemorrhage in ischemic stroke treated with intravenous alteplase: Safe implementation of treatments in stroke (sits) symptomatic intracerebral hemorrhage risk score. Stroke. (2012) 43:1524–31. 10.1161/STROKEAHA.111.644815 22442178

[7] Liu J Wang Y Jin Y Guo W Song Q Wei C. Prediction of hemorrhagic transformation after ischemic stroke: development and validation study of a novel multi-biomarker model. Front Aging Neurosci. (2021) 13:667934. 10.3389/fnagi.2021.667934 34122045 PMC8193036

[8] Spronk E Sykes G Falcione S Munsterman D Joy T Kamtchum-Tatuene J. Hemorrhagic transformation in ischemic stroke and the role of inflammation. Front Neurol. (2021) 12:661955. 10.3389/fneur.2021.661955 34054705 PMC8160112

[9] Butcher K Christensen S Parsons M De Silva DA Ebinger M Levi C. Post-thrombolysis blood pressure elevation is associated with hemorrhagic transformation. Stroke. (2010) 41:72–7. 10.1161/STROKEAHA.109.563767 19926841

[10] Dagonnier M Donnan GA Davis SM Dewey HM Howells DW. Acute stroke biomarkers: are we there yet? Front Neurol. (2021) 12:619721. 10.3389/fneur.2021.619721 33633673 PMC7902038

[11] Whiteley W Tian Y Jickling GC. Blood biomarkers in stroke: Research and clinical practice. Int J Stroke. (2012) 7:435–9. 10.1111/j.1747-4949.2012.00784.x 22463131

[12] Kamtchum-Tatuene J Jickling GC. Blood biomarkers for stroke diagnosis and management. Neuromolecular Med. (2019) 21:344–68. 10.1007/s12017-019-08530-0 30830566 PMC6722038

[13] Michetti F D'Ambrosi N Toesca A Puglisi MA Serrano A Marchese E. The s100b story: From biomarker to active factor in neural injury. J Neurochem. (2019) 148:168–87. 10.1111/jnc.14574 30144068

[14] Miki Y Gion Y Mukae Y Hayashi A Sato H Yoshino T. Morphologic, flow cytometric, functional, and molecular analyses of s100b positive lymphocytes, unique cytotoxic lymphocytes containing s100b protein. Eur J Haematol. (2013) 90:99–110. 10.1111/ejh.12036 23130680

[15] Steiner J Marquardt N Pauls I Schiltz K Rahmoune H Bahn S. Human cd8(+) t cells and nk cells express and secrete s100b upon stimulation. Brain Behav Immun. (2011) 25:1233–41. 10.1016/j.bbi.2011.03.015 21447379

[16] Yardan T Erenler AK Baydin A Aydin K Cokluk C. Usefulness of s100b protein in neurological disorders. J Pak Med Assoc. (2011) 61:276–81. 21465945

[17] Hill MD Jackowski G Bayer N Lawrence M Jaeschke R. Biochemical markers in acute ischemic stroke. CMAJ. (2000) 162:1139–40. 10789628 PMC1232364

[18] Zhou S Bao J Wang Y Pan S. S100β as a biomarker for differential diagnosis of intracerebral hemorrhage and ischemic stroke. Neurol Res. (2016) 38:327–32. 10.1080/01616412.2016.1152675 27078704

[19] Foerch C Wunderlich MT Dvorak F Humpich M Kahles T Goertler M. Elevated serum s100b levels indicate a higher risk of hemorrhagic transformation after thrombolytic therapy in acute stroke. Stroke. (2007) 38:2491–5. 10.1161/STROKEAHA.106.480111 17673718

[20] Rossi R Molina S Mereuta OM Douglas A Fitzgerald S Tierney C. Does prior administration of rtpa influence acute ischemic stroke clot composition? Findings from the analysis of clots retrieved with mechanical thrombectomy from the restore registry. J Neurol. (2021) 269:1913–20. 10.1007/s00415-021-10758-5 34415423 PMC8940807

[21] Fiorelli M Bastianello S von Kummer R Del Zoppo GJ Larrue V Lesaffre E. Hemorrhagic transformation within 36 hours of a cerebral infarct: relationships with early clinical deterioration and 3-month outcome in the European Cooperative Acute Stroke Study I (ECASS I) cohort. Stroke. (1999) 30:2280–4. 10.1161/01.STR.30.11.2280 10548658

[22] World Medical Association. World medical association declaration of helsinki: Ethical principles for medical research involving human subjects. JAMA. (2013) 310:2191–4. 10.1001/jama.2013.281053 24141714

[23] Adams HP Bendixen BH Kappelle LJ Biller J Love BB Gordon DL. Classification of subtype of acute ischemic stroke. Definitions for use in a multicenter clinical trial. Toast. Trial of org 10172 in acute stroke treatment. Stroke. (1993) 24:35–41. 10.1161/01.STR.24.1.35 7678184

[24] Fitzgerald S Rossi R Mereuta OM Jabrah D Okolo A Douglas A. Per-pass analysis of acute ischemic stroke clots: Impact of stroke etiology on extracted clot area and histological composition. J Neurointerv Surg. (2020). 10.1136/neurintsurg-2020-016966 33298510 PMC8606448

[25] Fitzgerald S Rossi R Mereuta OM Molina S Okolo A Douglas A. Large artery atherosclerotic clots are larger than clots of other stroke etiologies and have poorer recanalization rates. J Stroke Cerebrovasc Dis. (2021) 30:105463. 10.1016/j.jstrokecerebrovasdis.2020.105463 33242780 PMC7755299

[26] Rossi R Fitzgerald S Molina S Mereuta OM Douglas A Pandit A. The administration of rtpa before mechanical thrombectomy in acute ischemic stroke patients is associated with a significant reduction of the retrieved clot area but it does not influence revascularization outcome. J Thromb Thrombolysis. (2021) 51:545–51. 10.1007/s11239-020-02279-1 32936433 PMC7886731

[27] Rossi R Fitzgerald S Gil SM Mereuta OM Douglas A Pandit A. Correlation between acute ischaemic stroke clot length before mechanical thrombectomy and extracted clot area: impact of thrombus size on number of passes for clot removal and final recanalization. Eur Stroke J. (2021) 6:254–61. 10.1177/23969873211024777 34746421 PMC8564157

[28] Stritt M Stalder AK Vezzali E. Orbit image analysis: an open-source whole slide image analysis tool. PLoS Comput Biol. (2020) 16:e1007313. 10.1371/journal.pcbi.1007313 32023239 PMC7028292

[29] Douglas A Fitzgerald S Mereuta OM Rossi R O'Leary S Pandit A. Platelet-rich emboli are associated with von willebrand factor levels and have poorer revascularization outcomes. J Neurointerv Surg. (2020) 12:557–62. 10.1136/neurintsurg-2019-015410 31685695

[30] Krishnan R Mays W Elijovich L. Complications of mechanical thrombectomy in acute ischemic stroke. Neurology. (2021) 97:S115–s125. 10.1212/WNL.0000000000012803 34785610

[31] Hornig CR Dorndorf W Agnoli AL. Hemorrhagic cerebral infarction—a prospective study. Stroke. (1986) 17:179–85. 10.1161/01.STR.17.2.179 3515635

[32] Bozzao L Angeloni U Bastianello S Fantozzi LM Pierallini A Fieschi C. Early angiographic and ct findings in patients with hemorrhagic infarction in the distribution of the middle cerebral artery. AJNR Am J Neuroradiol. (1991) 12:1115–21. 1763737 PMC8331481

[33] Moulin T Crépin-Leblond T Chopard JL Bogousslavsky J. Hemorrhagic infarcts. Eur Neurol. (1994) 34:64–77. 10.1159/000117012 8174597

[34] Toni D Fiorelli M Bastianello S Sacchetti ML Sette G Argentino C. Hemorrhagic transformation of brain infarct: Predictability in the first 5 hours from stroke onset and influence on clinical outcome. Neurology. (1996) 46:341–5. 10.1212/WNL.46.2.341 8614491

[35] Lindley RI Wardlaw JM Sandercock PA Rimdusid P Lewis SC Signorini DF. Frequency and risk factors for spontaneous hemorrhagic transformation of cerebral infarction. J Stroke Cerebrovasc Dis. (2004) 13:235–46. 10.1016/j.jstrokecerebrovasdis.2004.03.003 17903981

[36] Wang X Tsuji K Lee SR Ning M Furie KL Buchan AM. Mechanisms of hemorrhagic transformation after tissue plasminogen activator reperfusion therapy for ischemic stroke. Stroke. (2004) 35:2726–30. 10.1161/01.STR.0000143219.16695.af 15459442

[37] Maier CM Hsieh L Crandall T Narasimhan P Chan PH. Evaluating therapeutic targets for reperfusion-related brain hemorrhage. Ann Neurol. (2006) 59:929–38. 10.1002/ana.20850 16673393

[38] Kaur J Zhao Z Klein GM Lo EH Buchan AM. The neurotoxicity of tissue plasminogen activator? J Cerebral Blood Flow Metabol. (2004) 24:945–63. 10.1097/01.WCB.0000137868.50767.E8 15356416

[39] Cuadrado E Ortega L Hernández-Guillamon M Penalba A Fernández-Cadenas I Rosell A. Tissue plasminogen activator (t-pa) promotes neutrophil degranulation and mmp-9 release. J Leukoc Biol. (2008) 84:207–14. 10.1189/jlb.0907606 18390930

[40] Lakhan SE Kirchgessner A Tepper D Leonard A. Corrigendum: Matrix metalloproteinases and blood-brain barrier disruption in acute ischemic stroke. Front Neurol. (2018) 9:202. 10.3389/fneur.2018.00202 29643832 PMC5893809

[41] Bourcier R Saleme S Labreuche J Mazighi M Fahed R Blanc R. More than three passes of stent retriever is an independent predictor of parenchymal hematoma in acute ischemic stroke. J Neurointerv Surg. (2019) 11:625–9. 10.1136/neurintsurg-2018-014380 30389897

[42] Donato R. S100: A multigenic family of calcium-modulated proteins of the ef-hand type with intracellular and extracellular functional roles. Int J Biochem Cell Biol. (2001) 33:637–68. 10.1016/S1357-2725(01)00046-2 11390274

[43] Selinfreund RH Barger SW Pledger WJ Van Eldik LJ. Neurotrophic protein s100 beta stimulates glial cell proliferation. Proc Natl Acad Sci U S A. (1991) 88:3554–8. 10.1073/pnas.88.9.3554 1902567 PMC51490

[44] Abraha HD Butterworth RJ Bath PM Wassif WS Garthwaite J Sherwood RA. Serum s-100 protein, relationship to clinical outcome in acute stroke. Ann Clin Biochem. (1997) 34:366–70. 10.1177/000456329703400405 9247667

[45] Barateiro A Afonso V Santos G Cerqueira JJ Brites D van Horssen J. S100b as a potential biomarker and therapeutic target in multiple sclerosis. Mol Neurobiol. (2016) 53:3976–91. 10.1007/s12035-015-9336-6 26184632

[46] Cristóvão JS Gomes CM. S100 proteins in alzheimer's disease. Front Neurosci. (2019) 13:463. 10.3389/fnins.2019.00463 31156365 PMC6532343

[47] Sathe K Maetzler W Lang JD Mounsey RB Fleckenstein C Martin HL. S100b is increased in parkinson's disease and ablation protects against mptp-induced toxicity through the rage and tnf-α pathway. Brain. (2012) 135:3336–47. 10.1093/brain/aws250 23169921 PMC3501971

[48] Juranek JK Daffu GK Wojtkiewicz J Lacomis D Kofler J Schmidt AM. Receptor for advanced glycation end products and its inflammatory ligands are upregulated in amyotrophic lateral sclerosis. Front Cell Neurosci. (2015) 9:485. 10.3389/fncel.2015.00485 26733811 PMC4686801

[49] Beer C Blacker D Bynevelt M Hankey GJ Puddey IB. Systemic markers of inflammation are independently associated with s100b concentration: results of an observational study in subjects with acute ischaemic stroke. J Neuroinflammation. (2010) 7:71. 10.1186/1742-2094-7-71 21034449 PMC2984413

[50] Weglewski A Ryglewicz D Mular A Juryńczyk J. Changes of protein s100b serum concentration during ischemic and hemorrhagic stroke in relation to the volume of stroke lesion. Neurol Neurochir Pol. (2005) 39:310–7. 16096936

[51] Montaner J Mendioroz M Delgado P García-Berrocoso T Giralt D Merino C. Differentiating ischemic from hemorrhagic stroke using plasma biomarkers: the s100b/rage pathway. J Proteomics. (2012) 75:4758–65. 10.1016/j.jprot.2012.01.033 22343074

[52] Foell D Wittkowski H Vogl T Roth J. S100 proteins expressed in phagocytes: a novel group of damage-associated molecular pattern molecules. J Leukoc Biol. (2007) 81:28–37. 10.1189/jlb.0306170 16943388

[53] de Haan JJ Smeets MB Pasterkamp G Arslan F. Danger signals in the initiation of the inflammatory response after myocardial infarction. Mediators Inflamm. (2013) 2013:206039 10.1155/2013/206039 24363498 PMC3864081

[54] Xia C Braunstein Z Toomey AC Zhong J Rao X. S100 proteins as an important regulator of macrophage inflammation. Front Immunol. (2018) 8:1908. 10.3389/fimmu.2017.01908 29379499 PMC5770888

[55] Niven J Hoare J McGowan D Devarajan G Itohara S Gannagé M. S100b up-regulates macrophage production of il1β and ccl22 and influences severity of retinal inflammation. PLoS ONE. (2015) 10:e0132688. 10.1371/journal.pone.0132688 26204512 PMC4512682

[56] Cheng M Su X Liu D Tian X Yan C Zhang X. Role of neutrophil-derived s100b in acute myocardial infarction patients from the han chinese population. Front Cardiovas Med. (2021) 7:595446. 10.3389/fcvm.2020.595446 33796567 PMC8008063

[57] Kuwar RB Stokic DS Leis AA Bai F Paul AM Fratkin JD. Does astroglial protein s100b contribute to west nile neuro-invasive syndrome? J Neurol Sci. (2015) 358:243–52. 10.1016/j.jns.2015.09.003 26382833

[58] Cai W Liu S Hu M Huang F Zhu Q Qiu W. Functional dynamics of neutrophils after ischemic stroke. Transl Stroke Res. (2020) 11:108–21. 10.1007/s12975-019-00694-y 30847778 PMC6993940

[59] Kang L Yu H Yang X Zhu Y Bai X Wang R. Neutrophil extracellular traps released by neutrophils impair revascularization and vascular remodeling after stroke. Nat Commun. (2020) 11:2488. 10.1038/s41467-020-16191-y 32427863 PMC7237502

[60] Rayasam A Hsu M Kijak JA Kissel L Hernandez G Sandor M. Immune responses in stroke: How the immune system contributes to damage and healing after stroke and how this knowledge could be translated to better cures? Immunology. (2018) 154:363–76. 10.1111/imm.12918 29494762 PMC6002204

[61] Kanamori M Endo T Shirakawa S Sakurai M Hidaka H. S-100 antigen in human t lymphocytes. Biochem Biophys Res Commun. (1982) 108:1447–53. 10.1016/S0006-291X(82)80069-7 6758780

[62] Stark K Massberg S. Interplay between inflammation and thrombosis in cardiovascular pathology. Nat Rev Card. (2021) 18:666–82. 10.1038/s41569-021-00552-1 33958774 PMC8100938

[63] Pennock ND White JT Cross EW Cheney EE Tamburini BA Kedl RM. Cell responses: naive to memory and everything in between. Adv Physiol Educ. (2013) 37:273–83. 10.1152/advan.00066.2013 24292902 PMC4089090

[64] Mukhopadhyay S Gabre J Chabasse C Bromberg JS Antalis TM Sarkar R. Depletion of CD4 and CD8 positive T cells impairs venous thrombus resolution in mice. Int J Mol Sci. (2020) 21:1650. 10.3390/ijms21051650 32121269 PMC7084232

[65] Onatsu J Vanninen R JÄkÄlÄ P Mustonen P Pulkki K Korhonen M. Tau, S100B and NSE as Blood Biomarkers in Acute Cerebrovascular Events. In Vivo. (2020) 34:2577–86. 10.21873/invivo.12075 32871787 PMC7652463

[66] Purroy F Farré-Rodriguez J Mauri-Capdevila G Vicente-Pascual M Farré J. Basal IL-6 and S100b levels are associated with infarct volume. Acta Neurol Scand. (2021) 144:517–23. 10.1111/ane.13487 34137020

