# Supplementary material for: S100b in acute ischemic stroke clots is a biomarker for post-thrombectomy intracranial hemorrhages
Source: Front Neurol. 2023 Jan 23;13:1067215. doi: 10.3389/fneur.2022.1067215 (PMC9900124; doi:10.3389/fneur.2022.1067215)
Supplement: Supplementary file 1 [file Table_1.docx]

**Table 1S. S100b expression in clots from cases with post thrombectomy intracranial hemorrhage (PTIH), divided by PTIH sub-type**

| **Type of haemorrhage** | **S100b expression (mm^2^)** | *Statistical analysis* |
| --- | --- | --- |
| Small petechial hemorrhagic infarction (HI1) | 0.28[0.02-1.31] | H5=3.435, P=0.633 |
| Confluent petechial hemorrhagic infarction (HI2) | 0.19[0.09-1.15] |  |
| Small parenchymal haemorrhage (PH1) | 0.60[0.22-1.01] |  |
| Large parenchymal haemorrhage (PH2) | 0.33[0.32-0.41] |  |
| Subarachnoid haemorrhage (SAH) | 0.44[0.28-0.71] |  |
| Subdural haematoma (SDH)^a^ | 0.02[0.02-0.02] |  |

Data are reported as median[IQ1-IQ3]. ^a^One case only
